# Graphene-Based Strategies in Liquid Biopsy and in Viral Diseases Diagnosis

**DOI:** 10.3390/nano10061014

**Published:** 2020-05-26

**Authors:** Annalaura Cordaro, Giulia Neri, Maria Teresa Sciortino, Angela Scala, Anna Piperno

**Affiliations:** 1 Department of Chemical, Biological, Pharmaceutical and Environmental Sciences, University of Messina, 98166 Messina, Italy; acordaro@unime.it (A.C.); nerig@unime.it (G.N.); mtsciortino@unime.it (M.T.S.); ascala@unime.it (A.S.); 2 Consorzio Interuniversitario Nazionale di ricerca in Metodologie e Processi Innovativi di Sintesi (C.I.N.M.P.I.S.), Unità Operativa dell’Università di Messina, 98166 Messina, Italy

**Keywords:** graphene, SERS, liquid biopsy, circulating tumor cells, exosomes, circulating nucleic acids, COVID-19

## Abstract

Graphene-based materials are intriguing nanomaterials with applications ranging from nanotechnology-related devices to drug delivery systems and biosensing. Multifunctional graphene platforms were proposed for the detection of several typical biomarkers (i.e., circulating tumor cells, exosomes, circulating nucleic acids, etc.) in liquid biopsy, and numerous methods, including optical, electrochemical, surface-enhanced Raman scattering (SERS), etc., have been developed for their detection. Due to the massive advancements in biology, material chemistry, and analytical technology, it is necessary to review the progress in this field from both medical and chemical sides. Liquid biopsy is considered a revolutionary technique that is opening unexpected perspectives in the early diagnosis and, in therapy monitoring, severe diseases, including cancer, metabolic syndrome, autoimmune, and neurodegenerative disorders. Although nanotechnology based on graphene has been poorly applied for the rapid diagnosis of viral diseases, the extraordinary properties of graphene (i.e., high electronic conductivity, large specific area, and surface functionalization) can be also exploited for the diagnosis of emerging viral diseases, such as the coronavirus disease 2019 (COVID-19). This review aimed to provide a comprehensive and in-depth summarization of the contribution of graphene-based nanomaterials in liquid biopsy, discussing the remaining challenges and the future trend; moreover, the paper gave the first look at the potentiality of graphene in COVID-19 diagnosis.

## 1. Introduction

Liquid biopsy is a minimally invasive technology for the detection of molecular biomarkers in blood and other body fluids (urine, saliva, ascites fluids, pleural effusions, etc.). The term was coined several decades ago, when was discovered, for the first time, the presence of extracellular nucleic acids in humans [1]; currently, it comprises not only the detection of extracellular/cell-free nucleic acids (NAs) with diagnostic significance but also of circulating tumor cells (CTCs) and extracellular vesicles (EVs), mainly exosomes (EXs). Although liquid biopsy cannot provide information related to tissue architecture and pathological microenvironment, it is considered a revolutionary technique that is opening unexpected perspectives in the early diagnosis and, in the therapy monitoring, severe diseases, ranging from cancer [2], metabolic syndrome [3], autoimmune disease [4], neurodegenerative disorders, and atherothrombosis [5] to prenatal screening [6].

Despite the high potential of liquid biopsy, the isolation, characterization, and quantification of NA, CTC, and EX biomarkers, due to their specific intrinsic features and low concentrations in the complex biological matrix, require complex procedures, and the systematic application in real practice is still hindered by many hurdles, such as unsatisfactory specificity and sensitivity, lack of standardization methods, and cost-effectiveness. Recently, a series of technological advancements in liquid biopsy has been obtained from the rapid development of nanotechnology-based strategies that provide a remarkable control over nanoparticle design, allowing to tailor their properties toward specific applications [7]. A plethora of nanomaterials, nanostructures, and molecular probes have been proposed for the fabrication of devices able to provide readable signals for early diagnosis and dynamic monitoring of diseases, taking advantage of their outstanding electrical, magnetic, optical, mechanical, or thermal characteristics at nanoscale dimensions [8]. Due to their unique physicochemical properties, arising from their high surface area, size, shape, unique optical properties, and surface chemistry, graphene-based materials (G) can realistically devise more advanced applications for liquid biopsy scope. The current review dealt with the recent advancements of G platforms for effectively capturing, identification, and quantification of NA, CT, and EX biomarkers. We discussed the main design criteria that have been used to develop multifunctional G platforms, bringing out the specific role of G in the selective capture and identification of heterogeneous biomarkers from the body fluids. Particular attention was reserved for the advances of liquid biopsy in cancer diagnosis and monitoring. Final remarks were devoted to challenges and the opportunity to adapt G technology for the diagnosis of emerging viral diseases, such as coronavirus disease 2019 (COVID-19).

## 2. Graphene Nanomaterials

The outcomes of graphene-based platforms in sensing applications are strictly correlated to the physicochemical properties of the starting material used for their fabrication [9]. However, a univocal classification of G broad family and their correlation with biosensing properties are challenging. Thus, the different synthetic approaches have been adopted for G preparation; the not homogeneous G nature (complexes structures with several oxidation states, varied lateral sizes, different number of layers, and different colloidal stability); the presence of impurities (often metal impurities); the formation of nanocomposites by a combination of G with organic or inorganic compounds have been taken in consideration for graphene-based biosensing applications [10].

G has been obtained by bottom-up or top-down approaches, differing for (i) the number and dimension of layers; (ii) the amount of oxygen functional groups scattered over the carbon surfaces; (iii) chemical features of compounds introduced during the post-synthetic decoration process, etc. [11,12,13,14].

Commonly, high-quality mono or multilayer G systems have been obtained by bottom-up approaches, such as epitaxial growth (EG) or chemical vapor deposition (CVD) on metallic substrates. These materials are endowed with ideal features (i.e., large surface area and high homogeneity) to be used as components of electronic devices. However, the high cost of these strategies, together with the requirement to transfer G on more suitable substrates, has limited the graphene’s scale-up production [15].

Top-down strategies, such as chemical or physical exfoliation of graphite bulk, are regarded as valuable synthetic options to develop G for diagnostic devices [16,17]. G commonly used in the biosensing field includes graphene oxide (GO), reduced graphene (G-red), functionalized graphene (*f*-G), together with emerging derivatives, such as graphene quantum dot (GQD), N-doped multiple graphene aerogel, graphene field-effect transistor (GFET) etc. The plethora of G is continuously supplied by new derivatives with unique properties, which potentially enable an entirely new generation of technologies beyond the limits of conventional materials [18,19,20].

GO is obtained by chemical oxidation of graphite and successive exfoliation of graphite oxide via ultrasonication. Oxygen functionality groups on GO surfaces are widely exploited in the chemical functionalization of GO, especially by esterification/amidation reactions at the carboxylic groups [21,22,23]. Processability and water stability due to ionizable groups on GO surfaces are the main advantages in the use of GO; whereas the structural defects on the sp^2^ network and the lacking electrical conductivity are the main limits for GO applications as an electronic device [24].

G-red is obtained from GO nanosheets by different techniques, including the solvothermal process or chemical reduction with hydrazine [25,26]. A partial restoring of the sp^2^ network, which results in an improved electrical conductivity and mechanical strength of G-red, compared to GO, has been obtained by the reduction process. Nowadays, G-red stable colloidal systems are obtained by using biocompatible reducing agents, such as gallic acid, starch, vitamin C, etc., allowing to reduce the cost and the environmental impact [27].

GQDs are fluorescent carbon nanosystems, generally arising from G or GO, composed of less than ten graphene layers with a later dimension less than 10 nm. GQDs do not possess only the intrinsic properties of graphene but also new properties due to edge effects and significant quantum confinement [28]. A wide variety of GQDs is obtained by bottom-up or top-down approaches. In the first case, the adopted strategies are characterized by a good size control and by the possibility to tune the GQDs properties on the basis of substrate nature. However, they suffer from some drawbacks, i.e., the employment of toxic solvent, high temperature, and substrate concentrations. Top-down approaches give a large scale production of GQDs due to the early synthetic steps and the use of cheap carbon starting materials [29].

GQDs have shown lower toxicity and higher photostability compared to other semiconducting quantum dots, and several applications, ranging from catalyst to nanomedicine, have been proposed. In particular, electrochemical, optical, and photoelectrochemical biosensors based on GQDs, characterized by a high sensing selectivity, have been developed [30].

An emerging class of 3D carbon materials (aerogel, foam, hydrogel, etc.) have been recently proposed for water decontamination and as conversion/storage energy devices [31]. Template-assisted methods, based on CVD strategy or graphene/GO layers assembling processes, such as self-assembly of G-red sheets reduced via the solvothermal or hydrothermal method, have provided 3D graphene-based materials, characterized by the intrinsic properties of G together with new interesting physicochemical properties, such as high porosity, low density, unique electrochemical performance [32]. N-doping strategies have been widely adopted to tune the electrochemical properties of G derivatives. N-doped G has shown high performance like photocatalytic systems for the reduction of CO_2_ and the degradation of organic contaminants under visible light [33].

The replacement of the traditional semiconductors-based electronic devices with a single layer graphene-based material has been proposed and used for the fabrication of GFET, proposed as sensors in physical, chemical, and biological application fields [34].

## 3. Tumor Biomarkers in Liquid Biopsy

**Figure 1:**
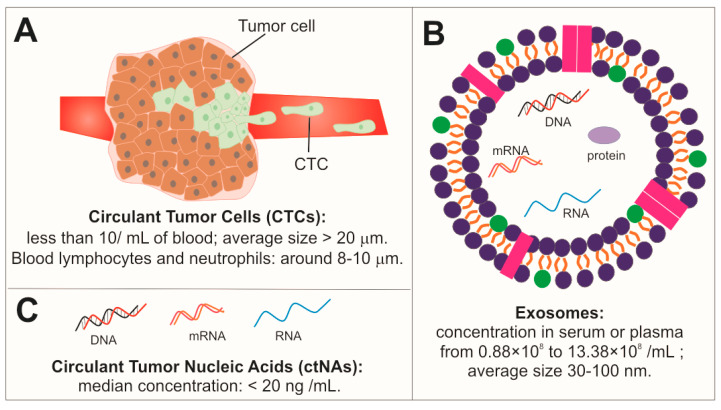
Schematic representation of typical cancer biomarkers of liquid biopsy: (**A**) Circulating tumor cells (CTCs); (**B**) Exosomes (EXs); (**C**) Circulant nucleic acids (ctNAs).

Considering the temporal and spatial heterogeneity and its evolution, the tumor needs to be monitored at distinct times of the disease for an efficient treatment. Therefore, there is an urgent need to search for minimally invasive approaches in order to detect and monitor the disease progression throughout the treatment. Indeed, surgical tissue biopsies are invasive procedures, often difficult to perform on organs that lie deep within the body, and their use is limited as they can give false-negative results due to sampling. Therefore, it is necessary to identify ideal biomarkers that can be used for the early diagnosis, detection of recurrence, and monitoring of metastasis for cancer. A liquid biopsy might be a promising approach because it deals with the communication in tumor microenvironment. According to several research studies, the liquid biopsy is defined as the capture and the analysis of tumor-related biomarkers in a fluid sample. The biomarkers are represented by circulant tumor cells (CTCs), circulant tumor nucleic acids (ctNAs), proteins, and/or tumor-derived extracellular vesicles (EVs), which have been shed from tumor masses (Figure 1) into the bloodstream, saliva, urine, cerebrospinal fluid (CSF), among other peripheral fluids of patients. The liquid biopsy provides a more comprehensive snapshot of intra-tumor clonal heterogeneity compared to single-site tissue biopsies and, in addition, can allow repeated blood sampling, thereby providing an insight into the evolutionary dynamics of cancer. For these reasons, liquid biopsy should be extensively studied due to its minimal invasiveness and can be used for the early diagnosis and monitoring of metastasis in cancer patients [35]. The main approaches to liquid biopsies have embraced the detection of CTCs [36,37], the capture of exosomes (EXs) that are secreted by tumor mass [38], and the analysis of ctDNA or miRNA in body fluid samples [39] since the first studies. Indeed, due to the rapid turnover of cancer cells and the constant release of tumor-derived nucleic acids, vesicles, and viable CTCs into the circulation, the ability to detect and characterize has enabled surgeons to analyze the evolution of the tumor at distinct times and, most importantly, in a non-invasive manner. Literature data have demonstrated that levels of these biomarkers increase in patients with several malignant types of tumors, such as breast, ovarian cancer, stomach, colorectal, prostate, lung, and others. However, most studies have been done in patients with late-stage cancer, mainly due to the considerably higher concentrations of the above-mentioned biomarkers in their blood. Based on these promising findings, data from Wroclawski and collaborators demonstrated that serum DNA levels were significantly increased in patients with colorectal cancer of stage IV and fluctuated during chemotherapy [40]. Lung cancer patients, if compared to the control patients, have demonstrated significant differences in ctDNA levels since stage I [41]. The fluctuations of ctDNA were proposed by Diehl et al. as a biomarker to monitor the course of therapy in patients with metastatic colorectal cancer (mCRC) undergoing surgery or chemotherapy treatments [42]. The level of ctDNA has been quantified by BEAMing (beads, emulsions, amplification, and magnetics) and compared to carcinoembryonic antigen marker (CEA), routinely used in the management of the disease in subjects with CRC [42]. Numerous gastrointestinal diseases can also lead to an increase in ctDNA, even if considered malignant or benign.

The diagnosis of ovarian cancer (OC) is mainly based on levels of biomarker CA-125 in blood and imaging. Recent data have shown that EVs possess advantages in terms of abundance, stability, and accessibility, compared with CTCs and ctDNA. Furthermore, the contents of EVs are tumor-specific and reveal a high correlation with tumor staging and prognosis [43]. Additionally, due to tumor heterogeneity, a panel of biomarkers will be more useful and reliable, instead of a single marker, for OC early diagnosis and screening high-risk individuals [44,45].

## 4. Circulant Tumor Cells (CTCs)

CTCs are a population of rare cancer cells detached from the primary tumor and shed into the bloodstream, becoming the main responsible for metastases in different organs. They are emerging as potential biomarkers and non-invasive alternative to tissue biopsy for the early detection, diagnosis, and prognosis of cancer, to improve the clinical settings of patients [46]. Since CTCs are extremely rare cells in the blood vessels (usually less than 10/mL of blood), their isolation from billions of red blood cells and millions of white blood cells and their accurate identification remain a challenge. Their heterogeneity (variety of surface protein expressions, sizes, and physical features), depending on the type and stage of cancer, makes laborious their isolation, requiring extremely sensitive and specific recognition methods [47]. In general, CTC detection includes four steps, such as capture, enrichment, detection, and the final release. The capture step is based on specific interactions between CTCs and materials (physical or antibody/antigen interaction). The enrichment step refers to CTCs isolation from the blood. The CTCs detection is generally carried out by fluorescence, surface-enhanced Raman scattering (SERS), or electrical impedance measurements. Finally, the enriched CTCs are released for further phenotype identification and molecular analyses [48]. To date, several technologies have been refined for CTCs detection, enrichment, and isolation, based on chemical or physical methods, such as capture by magnetic nanoparticles (NPs) [49], mechanical separation by size difference [50], microfluidic approaches [51,52], and immune-recognition methods [47].

Among the antibody-dependent isolation procedure based on specific biomarkers recognition, immunomagnetic technologies are often performed using anti-EpCAM antibody-functionalized magnetic NPs to specifically target EpCAM (Epithelial cell adhesion molecule) expressing cells. The epithelial cell adhesion molecule (EpCAM) is a transmembrane glycoprotein that mediates cell–cell adhesion in epithelial tissues. Since it has oncogenic potential, it has been extensively used for CTC capturing. To date, the only FDA approved CTC detection system is the CellSearch^®^ assay, although the high-cost fabrication limits its use. The kit is based on immunomagnetic separation, to target a specific antigen by using anti-EpCAM antibodies coupled to magnetic beads. The subsequent separation of the antigen-antibody complex can be achieved via exposure to a magnetic field [53].

The development of reliable, cost-effective, and sensitive CTC detection and isolation technologies plays a pivotal role in the early diagnosis and treatment of cancer. Nanomaterials offer excellent advantages to improve the sensitivity in biomolecule detection due to their high surface area to volume ratio and similar size with respect to biomolecules [54]. Many classes of nanomaterials have been incorporated into CTC research for highly sensitive and selective cell capture, i.e., magnetic and gold nanoparticles, carbon nanotubes, dendrimers, quantum dots, and graphene oxide (GO) [55]. Specifically, recent progresses in nanoscience have allowed designing nanoarchitectures based on multifunctional G platforms for the isolation and identification of CTCs, representing technological advancements in liquid biopsy [56]. In fact, the ease surface chemical modifications, together with its unique optical properties, make GO an attractive material for biomolecule detection [56], and the most commonly used strategies to isolate CTCs are based on traditional immunomagnetic separation, electrochemical technology, and microfluidic tools [47]. Because of the diamagnetic feature of all untreated biological materials, magnetic cell separation using bio-conjugated magnetic materials can be fruitfully applied to separate CTCs from whole blood, in a highly specific way, via targeted binding and subsequent separation using a bar magnet, avoiding light scattering and autofluorescence background from blood cells.

The combination of graphene oxide quantum dots (GOQDs) and magnetic nanoplatforms into a single nanoarchitecture functionalized with anti-GPC3 (Glypican 3) antibody has been proposed for the accurate identification and selective capture of liver cancer tumor CTCs [57]. An electrochemical sensing strategy based on aptamer-functionalized and gold nanoparticles array-decorated magnetic graphene nanosheet (AuNPs-Fe_3_O_4_-GS) has been reported for monitoring and capturing CTCs in human whole blood. The sensor takes advantage of the combination of two effects: the efficient recognition and capture of the target CTCs assured by selected aptamers and the signal amplification guaranteed by the functionalization of the gold nanoparticles (AuNPs) with electroactive species (6-ferrocenyl-1-hexanethiol or thionine) [58].

Several GO-based microfluidic devices have been proposed to enrich CTCs, based on their distinct biochemical properties toward other human blood components. Most of these devices focus on immunoaffinity-based technologies, which exploit specific antibodies widely expressed in cancer cells to isolate CTCs with high purity and sensitivity.

A microfluidic GO-based chip with accurate surface capture design has been reported for isolating CTCs from metastatic breast cancer patients, with high sensitivity and reproducibility. The use of GO as the base material for antibody conjugation enables the chip to detect CTCs from only 1 mL of blood, with high yield and reproducibility due to the high-density antibody presentation [59].

A microfluidic device exploiting immunocapture based on a tunable thermal-sensitive polymer-GO chip has been proposed for highly efficient capture and subsequent release of CTCs from breast and pancreatic cancer patients. The microfluidic device is coated with a composite film of functionalized GO dispersed in a thermoresponsive polymer matrix. The combination of a biocompatible GO, properly functionalized for immunocapture, with a thermosensitive polymer, has provided temperature-dependent modulation of capture/release, allowing the effective cell release for post-capture analysis. This device has overcome the common drawback of most immunoaffinity-based technologies reliant on antibodies attached to the capture surface, hindering the release of viable cells [60]. Electrochemical technology is also applicable to CTC recovery. A graphene-based electrochemical sensing platform, based on functionalized graphene-modified glassy carbon electrodes (GCEs), has been designed to be incubated with mammalian cells (i.e., different cancerous, multidrug-resistant cancerous, and metastatic human breast cells, as well as artificial CTC samples). The interactions with cell surface components, responsible for conjugating the target cells on the electrode surface, have been transduced to an ultrasensitive electrochemical response. The chemical diversity offered by the graphene probes has allowed discerning different cell surface/cell type, serving as a sensor array featuring selective receptors. The advantage of such an array-based sensing approach relies on the possibility to make an overall signature of CTCs, providing a fingerprint that allows for the classification and identification of cells [61]. A porous graphene-oxide (PGO) has been used to decorate light addressable-potentiometric-sensor (LAPS) surface, followed by the aptamer AS1411 anchoring (apta-PGO-LAPS), and is investigated as a light addressable potentiometric sensor. The CTC sensing interface has exploited the integration of electronic sensors with the robust and specific CTC’s bioprobe (aptamer). Specifically, the aptamer probe AS1411 has owned high binding affinity and specificity to the overexpressed nucleolin on the CTCs’ membrane [62].

A sensor, for clinical sample’s CTC detection, based on aptamer AS1411 functionalized graphene field-effect transistor (GFET) by using tetra (4-aminophenyl) porphyrin-mediated reduced GO as the channel material, has been recently proposed. The aptamer sensing strategy has been applied to isolate CTCs of human lung cancer cell line A549, breast cancer MDAMB-231, and cervical cancer HeLa, with good sensitivity [63].

A versatile super-sandwich cytosensor, based on GO-modified 3D microchip and Au-enwrapped silica nanocomposites (Si/AuNPs), fabricated by photolithography, has been developed as CTC-sensitive quantitative detection system. The sensor integrates two functional components: (1) an anti-EpCAM coating on GO for recognizing/capturing EpCAM-expressing cells, and (2) horseradish peroxidase (HPR) and anti-CA15-3 (Ab2) loaded in Si/AuNPs to improve the selectivity of target cells and amplify the electrochemical detection signal. The performance was assessed on MCF7 breast cancer cells, showing high sensitivity with a wide range of 10^1^ to 10^7^ cells mL^−1^ and a detection limit of 10 cells mL^−1^ [64]. A CTC isolation platform based on GO functionalized polyester fabric sheets bearing anti-EpCAM antibodies has been proposed as a low-cost, easy-to-fit, and disposable platform, assuring high sensitivity. Capture efficiency of 75–80% was obtained for cells with high EpCAM expressions [65].

A 3D hierarchical nanostructured graphene cell-captured foam with an anti-EpCAM coating (rGO/ZnO/anti-EpCAM foam) has been proposed for recognizing/capturing EpCAM-expressing cancer cells, showing some advantages compared to microfluidic-based devices, such as easy fabrication, increased cell-substrate contact frequency in all directions, microporosity, which allows normal red blood cells to travel through, but selectively captures CTCs, due to the anti-EpCAM coating. The performance of this 3D foam was investigated using EpCAM-positive cancer cell lines (MCF7, breast cancer cells), resulting in a cell-capture yield reaching up to 58% after an incubation time of 30 min [66].

For more comprehensive CTC enrichment, special attention must be focused on the choice of antibodies. By combining different antibodies in a single nanodevice, higher capture efficiency can be achieved than that obtained by single biomarker recognition. Reduced graphene oxide (rGO) films functionalized with anti-EpCAM and anti-prostate specific membrane antigen (anti-PSMA) antibodies have been recently fabricated by spray coating rGO solution onto a smooth glass slide. The antibody-modified rGO films exhibited a high efficiency (60%) of CTC capture from the blood of prostate cancer patients with prostate-specific antigen (PSA) levels of 4–10 ng mL^−1^ [67].

## 5. Exosomes (EXs)

EXs are a subgroup of cell-derived nano-sized (30–100 nm) extracellular vesicles (EVs) that have been recently recognized as new mediators for many cellular processes and potential biomarkers for non-invasive disease diagnosis and for monitoring treatment response, especially in cancer therapy. Mounting evidences have demonstrated the EX implication in several diseases, including viral pathogenesis [68], neurodegenerative diseases [69], and cancer growth and progression [70]. In particular, the release of EXs has been found to increase significantly in most neoplastic cells and occurs continuously at all stages of tumor development. Growing evidence has shown that the tumor-derived EXs carry characteristic proteins and RNAs in various cancer types, and the expression levels of these molecules are closely correlated with tumor progression [71]. Besides, the surface protein expression can provide invaluable information associated with the physiological states of the parental cells, that is why EXs are emerging as a novel disease diagnosis tool. Although EXs share several protein markers on their membrane, some of them are cell-specific and reflect the conditions of the secreting cell, meaning that there is a large heterogeneity among these biological markers in a single sample of withdrawn blood; this makes their isolation rather difficult. Up to date, most of the microfluidic devices are still not compatible with clinical analysis due to scalability, standardization, and validation. Further, several approaches are time-consuming, require extensive pre-treatment steps, and do not recover enough samples for genomic or proteomic analysis. Thus, there is a need for isolation techniques that selectively isolate EXs in a cost-effective and rapid manner [2]. Nevertheless, the performance of common isolation methods is significantly affected by contamination from other membrane-derived subcellular structures with high similarity in physical properties, resulting in very poor recovery yields. Numerous EX isolation techniques have been established so far, including ultracentrifugation, polymer-based precipitation, filtration, and affinity pull-down. Currently, the most common method for EX purification is the ultracentrifugation, which includes several centrifugation steps. Polymer-based precipitation relies on the formation of a polymer network to entangle all lipid components in the sample and to reduce their solubility for rapid removal under a low centrifugal force [72,73,74]. Membrane filtration has also been applied for size-based isolation of EXs, but EXs are prone to adhere to the filtration membranes, causing sample loss. Moreover, the additional force applied to pass the analyzed liquid through the membranes could potentially deform or damage the EXs [74]. Affinity pull-down is superior in selective separation of EXs using specific antibodies, but it requires large amounts of sample volumes.

**Figure 2:**
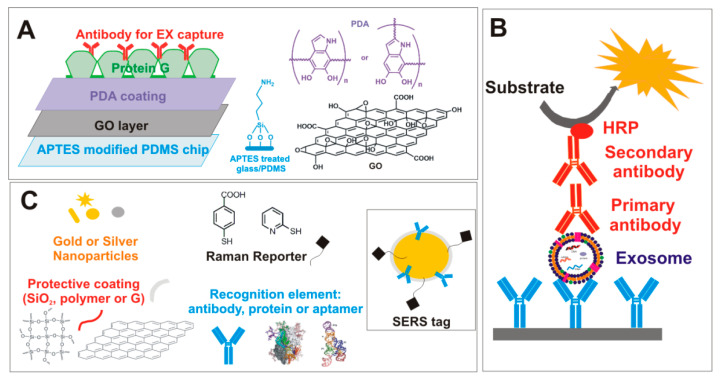
(**A**) Representative structure of the microfluidic platform based on graphene oxide/polydopamine (GO/PDA) for the immunocapture of exosome. (**B**) Representative EXs detection by ELISA colorimetric test using horseradish peroxidase (HRP) as a reporter enzyme in the secondary antibody. (**C**) Surface-enhanced Raman scattering (SERS) tag with related SERS tag toolbox.

The development of “bio-sensors” able to recognize EXs, without purification steps, from biological samples with very high accuracy and sensitivity, has recently spread among the scientific community. Generally, they combine the specificity of immunoaffinity-based systems with functionalized nanomaterials. Fang et al. [71] designed a hybrid platform that integrated two nanomaterials with different surface properties: the hydrophilic macroporous graphene foam (GF) and the amphiphilic periodic mesoporous organosilica (PMO). The high specific surface area of GF, after modification with the antibody against the EX protein marker, CD63 (specific exosome marker), allowed highly specific isolation of EXs from complex biological samples with high recovery. After lysis with methanol, the amphiphilic PMO was employed to rapidly recover the EX proteins, including the highly hydrophobic membrane proteins. Peptides obtained by protein digestion were analyzed by LC-MS/MS analysis (liquid chromatography-tandem mass spectrometry). Zhang et al. reported a microfluidic platform based on the graphene oxide/polydopamine (GO/PDA) system [75]. GO induced spontaneous polymerization of a 3D PDA surface coating, which was demonstrated to improve the efficiency of EX immuno-capture, suppressing the effects of non-specific adsorption. The platform was prepared by a layer-by-layer coating method (Figure 2), and the on-chip-captured EXs were detected by fluorescence analyses after the treatment with a mixture of biotinylated antibodies (CD63, CD81, and EpCAM). Streptavidin-conjugated β-galactosidase (S*β*G) was used as a reporter enzyme. The platform performance was proved in both molecular profiling and the quantitative EXs detection of purified samples from a colon cancer cell line or directly in plasma samples from ovarian cancer patients. Surface-enhanced Raman scattering (SERS) spectroscopy is a promising analytic tool for EXs’ ultra-detection. SERS’ biomedical applications include two general methodologies, called label-free detection and indirect approaches based on the use of a Raman reporter (RaR) linked to nobel NPs, commonly known as SERS tags or SERS-labeled NPs [76]. A SERS tag consists of four main components: (1) silver or gold NPs, which act as plasmonic enhancer; (2) Raman reporter (RaR) acting as fingerprint label; (3) a protective layer or coating shell that stabilizes the NPs, allowing the biomolecules grafting; (4) recognition moieties. Noble metal NPs inducing an enhanced electric field when LSPR (localized surface plasmon resonance) is excited by selected laser light sources [16] might be considered the SERS tag core. The RaR, an organic compound with a typical spectral fingerprint (i.e., benzenthiol, 4-mercaptobenzoic acid, etc.), ideally should cover the NPs to provide a stable, intense, and reliable Raman signal. The coating component of SERS-tag, although not essential, can improve the colloid stability and provide several advantages: (a) prevent the RaR leaching; (b) avoid contaminations; (c) reduce the intensity variations due to NP-NP interactions. Several protective coatings have been proposed, including biomolecules (i.e., bovine serum albumin), polymers (i.e., PEG), inorganic shell (i.e., SiO_2_), liposomes [76], and graphene [77]. In the last case, G acts as both a protective shell and RaR. Specific peptides, antibodies, or proteins are grafted in the external layer of the SERS-tag as recognition ligands of biomarkers [76].

## 6. Circulant Tumor Nucleic Acids (ctNAs)

Circulating tumor nucleic acids (ctNAs), such as circulating cell-free DNA, RNA, microRNA (Figure 1), represent an innovative tool for liquid biopsy applications [2,78,79]. The ctNA levels, compared to other circulating free biomarkers (such as CTCs), are detectable early in the bloodstream; therefore, they can be used for the initial tumor detection and the disease monitoring [80]. To date, several ctNA detection approaches based on fluorescence, SERS spectroscopy, radiochemical, enzymatic approach, chemiluminescence have been investigated [46,81,82]. Unfortunately, the detection of these biomarkers is challenging due to their small size and low concentrations in body fluids [80]. Different ultra-sensitive detection methods, including nucleic-acid sequence-based amplification [83], rolling circle amplification (RCA) [84], and polymerase chain reaction (PCR) [85], have been proposed. However, the complexity, the high cost of reagents and equipment, and the time-consuming protocols prevent the translation of these strategies in the market. To overcome some of these disadvantages, also the electrochemical approaches, characterized by a lower cost and a high detection sensitivity via signal amplification, have been proposed in the last years [86].

GO and rGO have been chosen as sensing platforms to detect circulating oligonucleotides and cells by fluorescent spectroscopy due to their ability to adsorb single-stranded (ss) oligonucleotides by noncovalent approaches (π-π interactions and/or hydrogen bonds) [87]. At the same time, GO and rGO have shown a lower affinity towards the double-stranded (ds) oligonucleotides due to poor accessibility of nucleobases inside the double helix and a lower ability to adsorb longer oligonucleotides due to lower diffusivity processes [88]. Moreover, G materials are able to almost completely quench the fluorescence emission of the fluorescent dye linked to ss oligonucleotide. In the presence of complementary target oligonucleotides (circulating oligonucleotides), the ss oligonucleotides marked with fluorescent dye can be released by G surface and complexed with the complementary target oligonucleotide, restoring the fluorescent emission, allowing the identification of the circulating oligonucleotide fragments.

A biosensing platform able to simultaneously detect and evaluate the amounts of miR-141 and miR-21 (two miRNAs overexpressed in the early and in the advanced stage of prostate cancer) from several body fluids (blood, urine, saliva) was investigated by Salih Hizir et al. [89]. The ability of GO to adsorb ss DNA on its surface and to quench fluorescence emission was exploited for the design of GO platform engineered with two fluorophore-labeled antisense DNA strands: fluorescein amidites (FAM)-labeled anti-miR-21 and Cy5-labeled anti-miR-141. The platform resulted in a fluorescent quenching at 520 nm (FAM channel) and 670 nm (Cy5 channel). In the presence of overexpressed miR-21, a fluorescent signal enhancement at 520 nm was observed, whereas overexpressed miR-141 induced a fluorescent signal increase at 670 nm. Non-target miRNAs induced only a lower fluorescent increase at these channels; therefore, the increase of fluorescent signal at 520 or 670 nm indicated the presence of miR-21 or miR-141, and the increase of intensity fluorescence signal level was used to determinate the concentration of miR-21 and miR-141 fragments. The system was proposed not only to detect prostate cancer disease but also to evaluate its advancement stage. Unfortunately, the low sensitivity and low specificity are typical problems of these nanodevices, hindering their clinical application [89].

A new GO-polymer-oligonucleotide (nGO-PEGMA/M2) and enzyme (*DNase I*) system able to detect miR-10b in an RNA pool taken from metastatic breast cancer cells were reported by Robertson et al. [90]. The insertion of a specific mismatch fragment into the probe sequence induced an increase of specificity towards miR-10b, an oligonucleotide overexpressed in breast cancer. The nGO-PEGMA/M2 *DNase I* system was able to distinguish miR-10b from miR-10a, which differed only for a single nucleotide. The presence of the endonuclease *DNase I* improved the fluorescent sensitivity of the probe but also the background fluorescent signal. To overcome this drawback, the edge of GO was functionalized with PEGMA, which hindered the access of *DNase I* on the GO surface to avoid the increase of fluorescence background signal due to undesired enzymatic activity [90].

The combination of the quenching properties of GO and cyclic enzymatic amplification method (CEAM) has allowed developing GO/ssDNA probes able to detect and discriminate among several mir-21 miRNAs in cell lysate media. The up-regulation expression of mir-21 miRNAs is involved in solid tumor growth. The biological media have been obtained from lung carcinoma cell line A-549 and mammary epithelial cells MCF-10A. The presence of complementary miRNA has induced the restoration of fluorescence due to miRNA/DNA complex formation, previously quenched by GO. Subsequently, miRNA released from the *DNase I* digestion can complex with another ssDNA probe on the GO surface to start another cycle, enhancing the fluorescent signal until all released ssDNA probes are completely consumed [91].

In the presence of divalent salt, GO is not able to discriminate between ssNAs and dsNAs [92]. On the contrary, rGO has shown a higher selectivity towards miRNA compared to GO in the same adsorption conditions [93]. Taking into account these findings, Yan et al. developed a magnetic system based on rGO (magnetic beads@APTES@rGO) able to selectively adsorb miRNA from the RNA pool isolated from healthy human plasma [88]. Magnetic beads were employed to obtain a faster extraction process by centrifugation. Moreover, in situ reverse transcriptions (RT), such as rolling circle amplification (RCA) strategy, were applied to desorb and detect miRNA by rGO surface [88].

Several challenges have been also focused on the detection of both circulating ss/ds DNA. Ruiyi et al. developed a nitrogen-doped multiple graphene aerogel/gold nanostar biosensor (N-doped MGA/GNS) able to detect dsDNA by human serum via electrochemical approach [94]. The hybrid N-doped MGA/GNS system showed an electrocatalytic activity towards Fe (CN)_6_^3−/4−^ improved in the presence of dsDNA, which was demonstrated by amperometric detection. The authors ascribed this behavior to the interaction between DNA and under-coordinated Au(I) sites bonded on the N-doped MGA-5 surface [94]. Another electrochemical biosensor composed of G decorated with Au nanorods and polythionine film (G/Au NR/PT) deposited onto glassy carbon electrode (GCE) was developed by Huang et al. for the detection of human papillomavirus (HPV) DNA in human serum [95]. G was used to enhance the surface area and the electric conductivity of the system; Au NRs (Au nanorods) were employed to increase the immobilization of DNA probe; polythionines were selected due to their good electron transfer ability and due to their ability to bond the Au NR surfaces by their amine groups. The thiolated capture probes (CP) were immobilized on the biosensor via electrostatic interactions and Au–S covalent bonds. CP was hybridized with one terminal of DNA target (TD), which arose from HPV-16 long terminal repeat sequences. Moreover, two auxiliary probes (AP) were developed to complex TD (fragment to be detected in human serum) by a long-range self-assembly process. Finally, the 1,10-phenanthrolineruthenium dichloride ([Ru(phen)_3_]^2+^) was used as an electrochemical indicator due to its ability to bond the DNA by electrostatic interactions. The increase of electrochemical response signal depended on the amount of ([Ru(phen)_3_]^2+^) bonded to DNA nanostructure. Worth noticing, the two AP sequences could bond with each other on the biosensor surface, giving rise to considerable lengthy self-assembled DNA nanostructure, only in the presence of TD [95].

## 7. Graphene-Based Strategies in the Diagnosis of Viral Diseases

Direct methods, exploiting graphene nanotechnology, for the rapid virus detection, have been only marginally investigated in the past, and no critical discussion has been reported in successive literature reviews [96,97].

This attitude was unchanging even during SARS–CoV-1 emergency that was responsible for the 2003 severe acute respiratory syndrome (SARS) infection in Asia, causing about 8000 cases and 774 deaths, also during the Middle East respiratory distress syndrome (MERS) of 2013, which affected Saudi Arabia causing close to 858 deaths [12,98,99,100]. Advances in nanotechnology have begun to play an important role in viral detection, to improve the detection limit, operational simplicity of viral diagnostics [78].

A coplanar-gate graphene field-effect transistors (GFETs) [71] have been proposed for the detection of HIV-1 (human immunodeficiency virus 1) and MLV (murine leukemia virus) viruses using antibodies of vesicular stomatitis Indiana virus (VSV) as biorecognition element. VSV antibodies are immobilized on the G layer using 1-pyrenebutanoic acid succinimidyl ester (PASE). PASE binds G by π-π interactions, anchoring the antibody’s primary amine groups by the opposite succinimidyl group. The formation of the virus-antibody complex leads to a downward shift of the Dirac point voltage, regardless of the types of detected viruses. The proposed platform has worked in a wide range of concentrations (from 47.8 aM to 10.55 nM), but the lack of virus specificity appears the main limitation of this strategy.

An surface plasmon resonance (SPR) sensor based on an polyamidoamine-functionalized rGO(composite, with monoclonal antibodies immobilized on self-assembled dithiobis (succinimidyl undecanoate, DSU) for the detection/quantification of Dengue virus (DENV), has been recently described [97].

The specificity and the sensibility of the sensor have been achieved by anchoring a stable biorecognition element (antibodies (IgM) against Dengue type 2 envelope proteins) on the gold surface of the sensor. The specific binding of antibody-DENV 2 E-protein allows a significant change in the angle of the reflectivity minimum that is correlated to Dengue virus detection. The proposed sensor has shown a sensitive and selective response towards DENV 2 E-proteins compared to DENV 1 E-proteins and ZIKV (Zika virus) E-proteins. Although no G materials have been integrated into the above-described sensor [97], the criteria used for its fabrication were included in this review since the strategy could be extended to other viruses, and the performance of SPR noble metal could be improved in the presence of G [76].

Differently from the past, the current sanitary pandemic emergency caused by the new type of coronavirus (SARS–CoV-2) is characterized by global effort to identify biomarkers that predict the severity of COVID-19 patients and to develop diagnostic tools for the rapid detection of SARS–CoV-2 infection [101].

Currently, nucleic acid testing on respiratory specimens is the reference gold standard method for the diagnosis of COVID-19 infected patients [102]. The test requires a series of laboratory procedures: (a) viral RNA extraction; (b) addition to a master mix containing nuclease-free water, reverse primers, a fluorophore-quencher probe, and a reaction mix (i.e., polymerase, reverse transcriptase, magnesium, nucleotides, and additives); (c) loading of extracted RNA/master mix into a PCR thermocycler; (d) several cycles at settled temperature. During the RT-PCR cycles, the cleavage of the fluorophore-quencher probe generates a fluorescent signal detected and recorded in real-time [101].

RT-PCR uses respiratory samples to genetically detect SARS–CoV-2; some data have suggested that 20–34% of COVID-19 patients resulted negative in the test despite being infected. This variance in the sensitivity could be mainly attributed to low viral load (i.e., patients tested in the early stage of the viral disease) [102]. Other RT-PCR issues include the time consuming and expensive analysis and the technical expertise in carrying out the text.

Other technologies, such as point-of-care technologies and serologic immunoassays, are rapidly emerging to address these deficiencies [78].

Analytic methods to assess prior infection and immunity to SARS–CoV-2 by antibody identification are essential for epidemiologic studies, although sensibility and specificity of the tests currently available in the market remain undefined. Cross-reactivity of antibody to non–SARS–CoV-2 coronavirus proteins is the main issue of these serologic tests [101,102]. The development of an antigen detection test [102] could take advantage of progress in the production of monoclonal antibodies against the nucleocapsid protein of SARS–CoV-2. The global effort to increase SARS–CoV-2 testing capacity takes advantage of the most recent advances in chemistry, molecular biology, genome technology, and nanotechnology. Several projects are ongoing in this direction, and some results are already reported in the literature [95,103].

The detection of SARS–CoV-2 in respiratory samples has been achieved by LSPR biosensor, combining the photothermal effect and plasmonic sensing transduction for SARS–CoV-2 viral nucleic acid [103].

**Figure 3:**
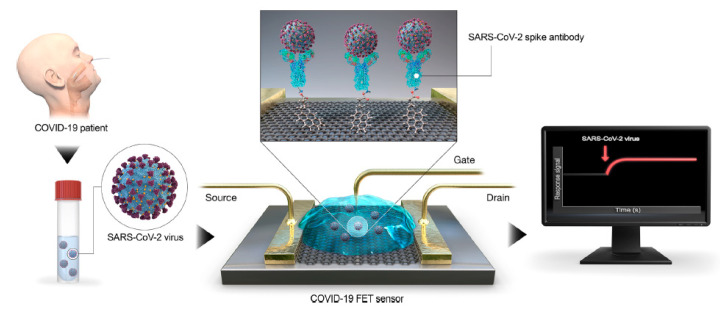
Coronavirus disease 2019 (COVID-19) field-effect transistor (FET)-sensor. Graphene is selected as sensing material and is decorated with the SARS–CoV-2 spike antibody using 1-pyrenebutanoic acid succinimidyl ester (PBASE) as interfacing molecule and probe linker. Reprinted with permission from reference [104], Copyright © 2020, American Chemical Society.

A field-effect transistor (FET)-based biosensing device for detecting SARS–CoV-2 spike protein (S) in clinical samples was reported by Seo et al. [104]. Antibodies against S protein were anchored to the graphene sheet (external coating of FET) by 1-pyrenebutanoic acid succinimidyl ester (PBASE, Figure 3).

The performance of the sensor is determined using antigen protein, cultured virus, and nasopharyngeal swab specimens from COVID-19 patients. The device could detect S protein at concentrations of 1 fg/mL in PBS and 100 fg/mL in the clinical transport medium, and it could distinguish the SARS–CoV-2 antigen protein from those of MERS-CoV. The successful fabrication of a COVID-19 FET sensor based on the integration of the SARS–CoV-2 spike antibody with graphene suggests the key role of G for diagnostic scope [80]. Specifically, the functionalization of G with diverse functional molecules [14,17,105,106] could be the key element to tailor its properties and to obtain advanced diagnostic tools for the SARS–CoV-2 diagnosis. Meanwhile, for the revision of this manuscript, some works dealing with sensors for COVID-19 diagnosis based on graphene are reported in the literature [107], and, although further researches are undoubtedly necessary, the leading role of G in the world’s fight against COVID-19 is clearly coming out [108].

In summary, the biomolecules till now used to target SARS–CoV-2 includes the viral RNA, the viral spike proteins, and the specific immunoglobulins produced by the host immune system. The biosensing community is actively working to improve portability, time, and cost of PCR-based SARS–CoV-2 detection, as well as to create manufacturable PCR-based microfluidic devices. Recently, also the gene-editing technology (CRISPR/Cas) has been developed to overcome the issues of PCR-based systems. Two different detection modes have been proposed in CRISPR technology, i.e., binding- or cleavage-based [109]. The sensor is developed by immobilization on a graphene-based field-effect transistor (GFET) of Cas9 with a sgRNA, specific to the target sequence of SARS COV-2; the electrical signal originated by the binding of the target nucleic acid by the Cas9–sgRNA complex is recorded via a simple handheld device without amplification.

## 8. Conclusions

The extraordinary properties of G make it a potential candidate to be routinely implemented in the design of biosensing platforms for liquid biopsy. Certainly, the innovation in diagnosis and monitoring of severe diseases could take advantage of the most recent progress in chemistry, molecular biology, genome technology, and nanotechnology. However, to give a significant contribution to the topics of great relevance for public health, such as cancer-fighting, neurodegenerative pathologies, emerging viral diseases, etc., the priority of collaborative research should be mainly focused on the opportunity to clinically translate the newly identified biomarkers using nanotechnology. Significant advancement has been achieved in the last years; however, data reproducibility remains the main drawback, and the selection of suitable nanomaterials for the development of devices is one of the key elements to obtain diagnostic tools that guarantee reproducible and reliable quantitative measurements.

Finally, the COVID-19 lesson indicates that the development of diagnostics is crucial to managing the pandemic outbreak, and certainly, G technology will assume a prominent role in the fabrication of innovative devices for the detection/quantification of viral nucleic acids/proteins actionable for detection at the point-of-care.
